# Can Pre-Treatment Inflammatory Parameters Predict the Probability of Sphincter-Preserving Surgery in Patients with Locally Advanced Low-Lying Rectal Cancer?

**DOI:** 10.3390/diagnostics11060946

**Published:** 2021-05-25

**Authors:** Richard Partl, Katarzyna Lukasiak, Bettina Stranz, Eva Hassler, Marton Magyar, Heidi Stranzl-Lawatsch, Tanja Langsenlehner

**Affiliations:** 1 Department of Therapeutic Radiology and Oncology, Comprehensive Cancer Center, Medical University of Graz, 8036 Graz, Austria; katarzyna.lukasiak@medunigraz.at (K.L.); bettina.stranz@klinikum-graz.at (B.S.); heidi.stranzl@medunigraz.at (H.S.-L.); tanja.langsenlehner@medunigraz.at (T.L.); 2 Division of Neuroradiology, Vascular and Interventional Radiology, Comprehensive Cancer Center Graz (CCC), Medical University of Graz, Auenbruggerplatz 9, 8036 Graz, Austria; eva.hassler@medunigraz.at (E.H.); marton.magyar@medunigraz.at (M.M.)

**Keywords:** low rectal cancer, locally advanced rectal cancer, predictive factors, pre-treatment parameters, inflammatory parameters sphincter-preserving surgery

## Abstract

There is evidence suggesting that pre-treatment clinical parameters can predict the probability of sphincter-preserving surgery in rectal cancer; however, to date, data on the predictive role of inflammatory parameters on the sphincter-preservation rate are not available. The aim of the present cohort study was to investigate the association between inflammation-based parameters and the sphincter-preserving surgery rate in patients with low-lying locally advanced rectal cancer (LARC). A total of 848 patients with LARC undergoing radiotherapy from 2004 to 2019 were retrospectively reviewed in order to identify patients with rectal cancer localized ≤6 cm from the anal verge, treated with neo-adjuvant radiochemotherapy (nRCT) and subsequent surgery. Univariable and multivariable analyses were used to investigate the role of pre-treatment inflammatory parameters, including the C-reactive protein (CRP), neutrophil-to-lymphocyte ratio (NLR), and platelet-to-lymphocyte ratio (PLR) for the prediction of sphincter preservation. A total of 363 patients met the inclusion criteria; among them, 210 patients (57.9%) underwent sphincter-preserving surgery, and in 153 patients (42.1%), an abdominoperineal rectum resection was performed. Univariable analysis showed a significant association of the pre-treatment CRP value (OR = 2.548, 95% CI: 1.584–4.097, *p* < 0.001) with sphincter preservation, whereas the pre-treatment NLR (OR = 1.098, 95% CI: 0.976–1.235, *p* = 0.120) and PLR (OR = 1.002, 95% CI: 1.000–1.005, *p* = 0.062) were not significantly associated with the type of surgery. In multivariable analysis, the pre-treatment CRP value (OR = 2.544; 95% CI: 1.314–4.926; *p* = 0.006) was identified as an independent predictive factor for sphincter-preserving surgery. The findings of the present study suggest that the pre-treatment CRP value represents an independent parameter predicting the probability of sphincter-preserving surgery in patients with low-lying LARC.

## 1. Introduction

Neo-adjuvant concomitant radiochemotherapy (nRCT) followed by surgery after an interval of several weeks has been established as the gold standard in the treatment of locally advanced rectal cancer (LARC) [1]. The use of nRCT has been associated with improved local tumor control as well as reduced toxicity when compared to the application of adjuvant RCT [2,3]. Furthermore, nRCT has been suggested to improve the possibility of sphincter-preserving surgery in low-lying LARC [4]. There is evidence indicating that a histopathological complete response (ypCR) after nRCT is an independent indicator for the sphincter-preserving surgery rate [4]. Additionally, ypCR has been associated with increased disease-free survival (DFS) and overall survival (OS) [5].

There is a growing body of data describing a relationship between blood-based surrogate parameters and the tumor response and outcome [5,6,7,8,9,10,11,12]. However, there have been very few attempts in the literature to analyze whether pre-treatment parameters can be used to predict the probability of sphincter-preserving surgery [4,13]. In a previously published study, we identified age, the relative lymphocyte value, and the interval between nRCT and surgery as independently associated with sphincter preservation [14].

Chronic inflammation has been shown to represent a pivotal contributor to the development and progression of a variety of cancers [15,16]. Previously, various studies have shown significant associations between blood-based inflammatory parameters such as C-reactive protein (CRP), the neutrophil-to-lymphocyte ratio (NLR), and the platelet-to-lymphocyte ratio (PLR) and prognosis in several cancer entities, including rectal cancer [17,18,19,20,21,22,23,24,25]. However, to the best of our knowledge, data on the predictive role of inflammatory parameters on the sphincter-preservation rate are currently not available.

Hence, the aim of the present cohort study was to elucidate the predictive role of pre-treatment inflammatory biomarkers for sphincter-preserving surgery and provide data on the prognostic outcome in a large European cohort of patients with low-lying rectal cancer consistently treated with nRCT.

## 2. Materials and Methods

In this cohort study, a total of 848 consecutive patients with histologically verified LARC, who were referred for radiotherapy from 2004 to 2019 at the Department of Therapeutic Radiology and Oncology, were retrospectively reviewed. Patients with low-lying LARC who had undergone nRCT and subsequent surgical resection were eligible for the present study. Patients who received additional induction chemotherapy or had a premature termination of radiation therapy were excluded from further analysis. A total of 363 patients met the inclusion criteria and were further analyzed.

Pretreatment colonoscopy, rigid proctoscopy, digital rectal examination, endorectal ultrasound, and pelvic computed tomography (CT) or magnetic resonance imaging (MRI) were performed to determine clinical tumor stage (cT) and clinical lymph node involvement. In order to rule out distant metastases, a thoracic and an abdominal CT were performed.

Concomitant chemotherapy consisted either of continuous intravenous infusion with 5-fluoruracil (1000 mg/m^2^) administered during the first and last week of radiotherapy or of an oral dose of capecitabine (1700 mg/daily) on each day of radiation treatment. Radiotherapy was planned and administered in a consistent manner throughout the study period. To exclude the small bowel, an open tabletop device (belly-board) was used when positioning the patient for the planning CT, and the perineum was marked with a radiopaque marker. Before CT, all patients were given an oral contrast agent to visualize the small bowel. All patients received radiotherapy in a 3D-conformal 3- or 4-field technique with photon energies of 6 or 18 MEV up to a total dose of 45–46 Gy in 23–25 fractions of 1.8 or 2 Gy (5 days/week). After a median interval of 6.6 weeks, either a total mesorectal excision (TME) or an abdominoperineal rectum resection (APR) was performed.

The following baseline parameters, which were documented prior to nRCT, were extracted from the medical charts: patient age at initiation of nRCT, sex, smoking, body mass index (BMI), Karnofsky performance status, cT, clinical lymph node involvement, histopathological subtype, histopathological tumor grading, and a full blood profile (erythrocytes, leucocytes, hemoglobin, thrombocytes, neutrophils, granulocytes, lymphocytes, serum lactate dehydrogenase (LDH), CRP, NLR, PLR). In accordance with the current standards for CRP determination of the Clinical Institute of Medical and Chemical Laboratory Diagnostics, Medical University of Graz, a plasma CRP level of ≥5 mg/L was considered pathological and selected as the cut-off value for analysis. In accordance with previously published studies, a cut-off value of >3 was used for the NLR, whereas the PLR was categorized into three groups (<150, 150–300, and >300) [26,27,28].

Clinical follow-up was conducted by the referring surgeon in accordance with institutional recommendations and by the Department of Therapeutic Radiology and Oncology. Clinical examination, proctoscopy/colonoscopy, and abdominal ultrasound were performed twice a year (years 1–2) and once a year (years 3–5). Additional imaging was performed if indicated.

### Statistical Analyses

Data are presented as mean values, and standard deviation or median values and range are reported for continuous data; absolute numbers are reported, and relative frequencies are provided for categorical data. The relationship between clinical parameters and sphincter-preserving surgery was first analyzed using univariable logistic regression analysis. A stepwise multivariable logistic regression analysis was then performed including all variables that showed a *p*-value of ≤0.2 in the univariable analysis. In addition, local recurrence-free survival (RFS), cancer-specific survival (CSS), and overall survival (OS) were calculated using Kaplan–Meier analysis, and log-rank tests were applied for statistical comparisons between curves. The RFS was calculated as the time from the start of radiotherapy to the development of local recurrence; the CSS was defined from the initiation of radiotherapy to the date of cancer-related death. The OS was defined as the time from the start of treatment to the date of death of any cause. All the statistical analyses were performed using the Statistical Package for Social Sciences version 25.0.0 (SPSS Inc., Chicago, IL, USA). A two-sided *p*-value < 0.05 was considered statistically significant.

The study complied with the Declaration of Helsinki and was performed in accordance with national law. The study protocol has been approved by the local Ethical Committee. As this was a retrospective non-interventional study, the institutional review board waived the need for written informed consent from the participants.

## 3. Results

A total of 363 patients were included in the present analysis. Patient characteristics are presented in Table 1. Median age at start of nRCT was 66.7 years (mean 65.3 ± 10.9).

The mean NLR (calculated as the absolute neutrophil count divided by the absolute lymphocyte count) and PLR (calculated as the absolute platelet count divided by the absolute lymphocyte count) were 3.5 ± 1.8 and 191.2 ± 91.0, respectively. The mean plasma CRP level was 8.0 ± 17.7 mg/L.

In 103 patients (30.7%), the CRP value was >5 mg/L; in 232 patients (69.3%), a CRP value ≤ 5 mg/L was detected. In 195 patients (53.7%), the NLR was >3, while in 168 patients (46.3%), a NLR ≤ 3 could be observed. Furthermore, there were 121 patients (34.9%) with a PLR < 150, 191 patients (55%) with a PLR 150–300, and 35 patients (10.1%) with a PLR > 300.

A TME was performed in 210 out of 363 patients (57.9%); in the remaining 153 patients (42.1%), an APR was performed. A complete histopathological response (ypT0 ypN0) was found in 59 out of 363 patients (16.3%). No association between complete tumor response and TME rate (*p* = 0.258) was detected.

### 3.1. Baseline Patient and Tumor Parameters Associated with Sphincter-Preserving Surgery

In univariable analyses, patient age (OR = 1.845, 95% CI: 1.181–2.884, *p* = 0.007), clinical T-size (OR = 2.769, 95% CI: 1.465–5.233, *p* = 0.002), and CRP value (OR = 2.548, 95% CI: 1.584–4.097, *p* < 0.001) were significantly associated with the sphincter-preserving surgery rate, whereas pre-treatment NLR (OR = 1.098, 95% CI: 0.976–1.235, *p* = 0.120) and PLR (OR = 1.002, 95% CI: 1.000–1.005, *p* = 0.062) was not significantly associated with the type of surgery. The results of univariable analysis are shown in Table 2.

In multivariable analyses, the pre-treatment CRP level remained a significant predictor for sphincter-preserving surgery (OR = 2.544, 95% CI: 1.314–4.926, *p* = 0.006). Additionally, the age at the start of irradiation (OR = 2.475, 95% CI: 1.249–4.903, *p* = 0.009) and clinical T-size (OR = 3.759, 95% CI: 1.214–11.641, *p* = 0.022), as well as tumor grade 3 (OR = 14.067, 95% CI: 1.896–104.376, *p* = 0.010), were significantly associated with the rate of sphincter-preserving surgery. The results of the multivariable analysis are given in Table 3.

### 3.2. Outcome by Type of Surgery

After a median follow-up time of 52 months (range, 1.1–161 months; mean, 60 months), 27 patients (7.4%) developed a local recurrence and 34 patients (9.4%) died due to cancer-related progression. A total of 50 patients (13.8%) died of any cause. The 3- and 5-year Kaplan–Meier estimates for local recurrence free-survival (RFS) were 92.6% and 90.8%, respectively; the 3- and 5-year estimates for cancer-specific survival (CSS) were 93.4% and 89.9%, respectively; and the 3- and 5-year overall survival (OS) estimates were 91% and 86%, respectively.

**Figure 1:**
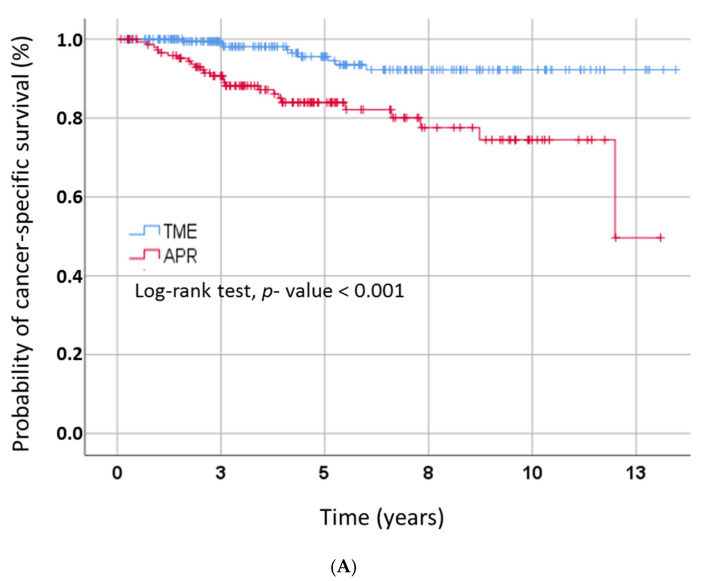

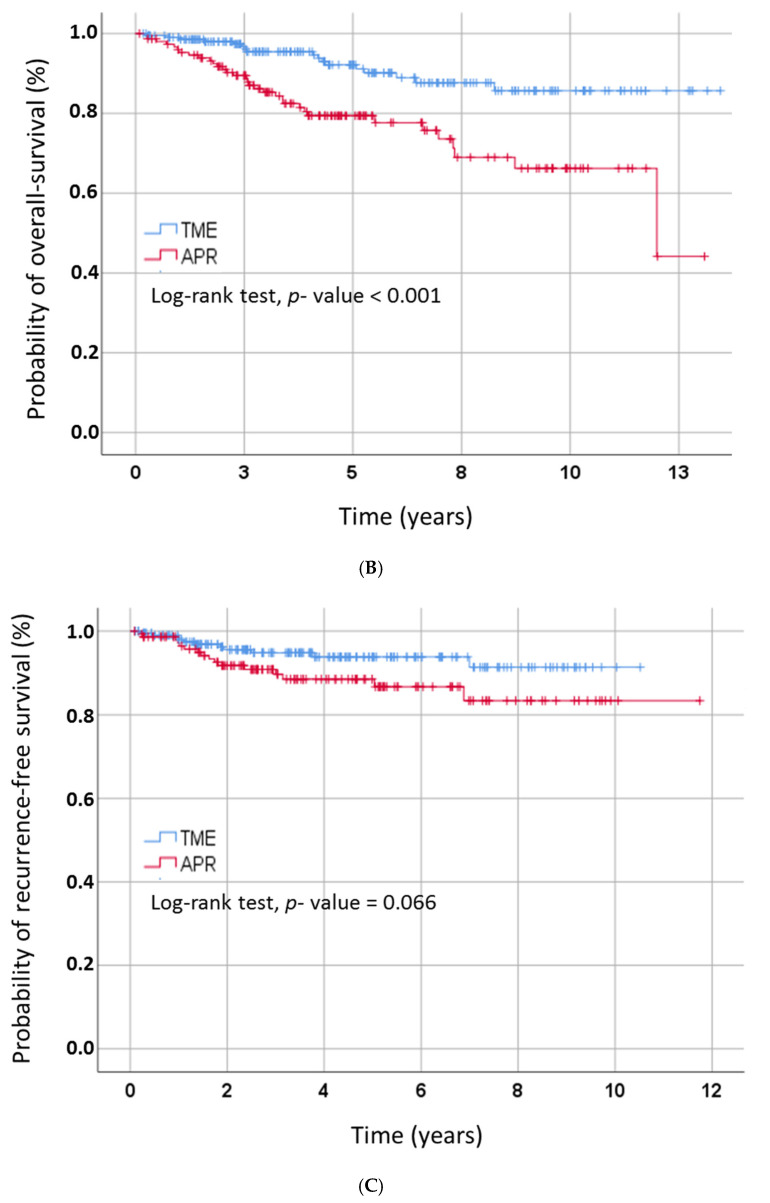
(**A**) Kaplan–Meier curves for cancer-specific survival by type of surgical treatment. Abbreviations: APR, abdominoperineal rectum resection; TME, total mesorectal excision; (**B**) Kaplan–Meier curves for overall survival by type of surgical treatment. Abbreviations: APR, abdominoperineal rectum resection; TME, total mesorectal excision; (**C**) Kaplan–Meier curves for recurrence-free survival by type of surgical treatment. Abbreviations: APR, abdominoperineal rectum resection; TME, total mesorectal excision.

Among patients treated with sphincter-preserving surgery, a significantly increased CSS (*p* < 0.001) as well as OS (*p* < 0.001) were observed when compared to patients who had undergone APR (Figure 1A,B). Kaplan–Meier estimates of CSS rates at 3 and 5 years were 98.1% and 94.5%, respectively, after sphincter-preserving surgery, compared to 87.2% and 82.1% after APR. Estimated OS rates at 3 and 5 years were 95.5% and 91.2%, respectively, after sphincter-preserving surgery, and 85.3% and 77.7% after APR, respectively.

For local RFS, no significant difference between patients treated with sphincter-preserving surgery and those treated with APR was detected (*p* = 0.066; Figure 1C).

## 4. Discussion

In previous years, several clinical parameters affecting surgical procedures in rectal cancer patients have been identified. Currently, it is widely accepted that the distance of the tumor from the anal verge represents an important predictor of sphincter-preserving surgery [4,13,29,30,31,32]. Another widely accepted factor influencing the surgical procedure is the experience of the surgeon. Several studies have shown an association between surgeon caseload and sphincter preservation. Furthermore, in centers with special expertise in colorectal cancer surgery, high rates of TME can be observed [32,33]. However, APR is still indicated in patients with tumor infiltration to the external anal sphincter and levator muscles.

The effect of nRCT on sphincter-preserving surgery is a controversial topic currently being discussed. Various studies have found no significant differences in the APR rate between nRT with subsequent surgery and primary surgery or between nRT and nRCT with subsequent surgery [34,35]. However, it has been shown that a good response to nRCT increases the rate of sphincter-preserving surgeries in patients with distal tumor localization. Crane et al. reported a higher sphincter-preserving surgery rate after clinical complete response (cCR) following nRCT in patients with a distal tumor location within ≤3 cm of the anal verge [4]. The probability of a sphincter-preserving procedure was twice as high for patients with cCR as that for those without cCR (44% vs. 22%; *p* = 0.01). A good tumor response to nRCT has also been associated with improved local tumor control and OS [5]. However, approximately 40% of patients show no or only a small tumor response to nRCT [36].

In low-lying rectal cancer, data on predictive parameters affecting the type of surgical procedure are still very sparse. In a prior study by our group, we identified patient age, the relative lymphocyte value, and the interval between nRCT and surgery as independently associated with sphincter preservation in low-lying rectal cancer [14]. Data on inflammatory parameters affecting surgical procedure cannot currently be found in the literature. To the best of our knowledge, to date, our observational study is the first to focus on the predictive role of inflammatory parameters including the pre-treatment NLR, PLR, and CRP value on sphincter preservation in low-lying LARC. In a total of 363 patients treated with nRCT, we were able to show a significant relationship between the pre-treatment plasma CRP level and sphincter-preserving surgery rate. Furthermore, we observed that the pre-treatment clinical T-size and the tumor grade were significantly associated with the type of surgical procedure, whereas no significant relationship between pre-treatment NLR and PLR and the type of surgical procedure was detected.

C-reactive protein (CRP) is an acute-phase protein primarily produced in response to systemic inflammation that plays an important role in the development and progression of a variety of cancers due to the upregulation of various cytokines and pro-angiogenic factors [15,37,38,39]. The expression of CRP is influenced by different cytokines and cytotoxic factors that are linked to cancer cell proliferation, growth, and migration [19,20]. Thus, CRP may represent a sensitive surrogate marker of mediators contributing to cancer cell growth and migration [22]. In rectal cancer, Kim et al. observed an association between elevated CRP levels and cancer-specific survival. Furthermore, elevated CRP was associated with poorer tumor regression [40]. Singh et al. summarized the value of serum CRP in predicting anastomotic leak after colorectal surgery in a meta-analysis with a total of 2483 patients [41].

Recently, inflammatory biomarkers such as NLR and PLR have been proposed as useful prognostic parameters in various cancer entities [42,43,44]. However, in rectal cancer, the prognostic significance of these parameters is still debatable. Although a number of studies have shown the prognostic significance of NLR and PLR, several studies did not identify any associations with survival outcomes [45,46,47,48]. Similarly, we were unable to detect a significant relationship between NLR or PLR and the type of surgical procedure in our cohort.

Recently, various biomarkers have been proposed for the early detection, tumor recurrence, and prognosis of CRC [49]. Through the use of liquid biopsy, circulating tumor cells, circulating tumor DNA, microRNA, cell-free DNA (cfDNA), and exosomes can be obtained, which represent promising components for prognostic and predictive purposes. Circulating tumor cells in the bloodstream have been identified as an unfavorable prognostic parameter in several cancer types. Plasma cfDNA are DNA fragments released by both normal and tumor cells. KRAS and BRAF mutation status in plasma cfDNA are highly correlated with mutation status in tumor tissue [50]. Early identification of these mutations via liquid biopsy can also be used to identify drug resistance against therapeutic agents and may enable more tailored treatment [51]. The analysis of cfDNA fragments represents another approach to predicting treatment response. According to Agostini et al., the ratio of tumor cfDNA fragments and physiological cfDNA is a promising marker of the tumor response after nRCT [52].

Currently, there is also an increasing amount of interest in the development of sensitive and specific noninvasive screening methods to identify patients with colorectal cancer (CRC). For instance, Altomare et al. evaluated the reliability of a breath test for the diagnosis of CRC and were able to demonstrate that the analysis of exhaled volatile compounds discriminated between cancer patients and healthy controls [53].

However, some limitations of the present study have to be taken into account. Due to the retrospective nature of the present study, we cannot completely rule out a selection bias or unequally distributed unknown clinicopathological confounders that may have caused bias in the observed results. Furthermore, the distance between the lower tumor margin and the anal verge is an important independent factor for the determination of a sphincter-preserving procedure. We have decided not to incorporate these data in our analysis due to conflicting results between pre-treatment CT, MRT, and rigid proctoscopy. The surgeons’ experience is a relevant confounder affecting the sphincter-preservation rate that we could not account for. Finally, CRP is a nonspecific marker of inflammation and might be influenced by several conditions, such as bacterial or viral infection, chronic autoimmune disease, severe stress, and surgical treatments.

Nevertheless, even considering these limitations, our data support the hypothesis that the pre-treatment CRP level might represent an independent predictive factor for sphincter-preserving surgery in patients with low-lying LARC. If validated in further studies, determination of the pre-treatment CRP level could be a relevant additional component in clinical practice in order to estimate the potential surgical procedure and to provide a more tailored cancer treatment.

## 5. Conclusions

The pre-treatment CRP level seems to impact the probability of a sphincter-preserving procedure significantly in patients with low-lying LARC and may support oncological therapy decisions. The CRP level might serve as a readily available and inexpensive predictive parameter that could be useful in daily oncologic clinical practice and help to identify patients who may benefit from a more aggressive neo-adjuvant treatment strategy. However, further large-scale prospective studies are warranted to confirm and extend our findings.

## Figures and Tables

**Table 1:** Summary of baseline patient characteristics.

Parameter	*n* (Missing %)	*n* (%) * or Median Value (Mean ± SD)
Sex	363 (0%)	
Male		249 (68.6%)
Female		114 (31.4%)
Age (years)	363 (0%)	
≤60		131 (36.1%)
>60		232 (63.9%)
Smoking	316 (12.9%)	
No		268 (84.8%)
Yes		48 (15.2%)
Karnofsky performance status	234 (35.5%)	
≤80%		20 (8.5%)
>80%		214 (91.5%)
Clinical T-size	363 (0%)	
cT 1/2		23 (6.3%)
cT 3		293 (80.7%)
cT 4		47 (13.0%)
Clinical nodal involvement	363 (0%)	
cN0		153 (42.1%)
cN+		210 (57.9%)
Clinical stage	363 (0%)	
I		12 (3.3%)
II		141 (38.8%)
III		210 (57.9%)
Histopathological subtype	363 (0%)	
Adenocarcinoma		340 (93.7%)
Adenocarcinoma (mucinous)		23 (6.3%)
Tumor grade	362 (0.6%)	
G1		24 (6.6%)
G2		312 (86.2%)
G3		26 (7.2%)
Erythrocyte count (T/l)	357 (1.7%)	4.6 (4.6 ± 0.6)
Leucocyte count (G/l)	358 (1.4%)	7.2 (7.7 ± 4.6)
Hemoglobin	357 (1.7%)	13.7 (13.4 ± 1.9)
Thrombocyte	358 (1.4%)	266 (279.8 ± 92.3)
Absolute neutrophil value	345 (4.9%)	4.8 (5.1 ± 1.8)
Absolute lymphocyte value	348 (4.1%)	1.5 (1.6 ± 0.5)
CRP value (mg/L)	335 (7.7%)	
≤5		232 (69.3%)
>5		103 (30.7%)
Neutrophil-to-lymphocyte ratio	343 (5.5%)	
≤3		168 (46.3%)
>3		195 (53.7%)
Platelet-to-lymphocyte ratio	347 (4.4%)	
<150		121 (34.9%)
150–300		191 (55.0%)
>300		35 (10.1%)
Radiation dose (fraction/total)	363 (0%)	
1.8/45 Gy		91 (25.1%)
2/46 Gy		272 (74.9%)
Chemotherapy	363 (0%)	
5-Fluorouracil		267 (73.6%)
Capecitabine		96 (26.4%)

* Percentages are calculated by referring only to patients without missing values. Abbreviations: LDH, lactate dehydrogenase; CRP, C-reactive protein; SD, standard deviation.

**Table 2:** Patient and tumor parameters: results of univariable analysis.

Parameter	Sphincter Preservation, n (%) or Mean Value ± SD	Abdominoperineal Resection, n (%) or Mean Value ± SD)	*p*-Value
Sex			0.167
Male	138 (65.7%)	111 (72.5%)	
Female	72 (34.3%)	42 (27.5%)	
Age (years)			0.007
≤60	88 (41.9%)	43 (28.1%)	
>60	122 (58.1%)	110 (71.9%)	
Smoking			0.311
No	152 (83.1%)	116 (87.2%)	
Yes	31 (16.9%)	17 (12.8%)	
Karnofsky performance status			0.149
≤80%	8 (6.2%)	12 (11.5%)	
>80%	122 (93.8%)	92 (88.5%)	
Clinical T-size			0.002
cT 1–3	193 (91.9%)	123 (80.4%)	
cT 4	17 (8.1%)	30 (19.6%)	
Clinical nodal involvement			0.912
cN0	88 (41.9%)	65 (42.5%)	
cN+	122 (58.1%)	88 (57.5%)	
Clinical stage			0.912
I/II *	88 (41.9%)	65 (42.5%)	
III	122 (58.1%)	88 (57.5%)	
Histopathological subtype			0.066
Adenocarcinoma	201 (95.7%)	139 (90.8%)	
Adenocarcinoma (mucinous)	9 (4.3%)	14 (9.2%)	
Tumor grade			0.054
G1	16 (7.7%)	8 (5.3%)	
G2	184 (88.0%)	128 (84.2%)	
G3	9 (4.3%)	16 (10.5%)	
Erythrocyte count (T/l)	4.7 ± 0.5	4.6 ± 0.8	0.327
Leucocyte count (G/l)	7.8 ± 5.8	7.6 ± 2.3	0.793
Hemoglobin	13.6 ± 1.8	13.2 ± 1.9	0.040
Thrombocyte	276 ± 93	284 ± 90	0.407
Absolute neutrophil value	4.9 ± 1.7	5.2 ± 1.9	0.126
Absolute lymphocyte value	1.6 ± 0.5	1.6 ± 0.6	0.276
CRP value (mg/L)			<0.001
≤5	152 (77.6%)	80 (57.65)	
>5	44 (22.4%)	59 (42.4%)	
Neutrophil-to-lymphocyte ratio			0.061
≤3	106 (50.5%)	62 (40.5%)	
>3	104 (49.5%)	91 (49.5%)	
Platelet-to-lymphocyte ratio			0.114
<150	78 (38.6%)	43 (29.7%)	
150–300	108 (53.5%)	83 (57.2%)	
>300	16 (7.9%)	19 (13.1%)	
Radiation dose (fraction/total)			0.740
1.8/45 Gy	54 (25.7%)	37 (24.2%)	
2/46 Gy	156 (74.3%)	116(75.8%)	
Chemotherapy			0.911
5-Fluorouracil	154 (73.3%)	113 (73.9%)	
Capecitabine	56 (26.7%)	40 (26.1%)	

* Because there were only 12 stage I tumors, stage I and II were grouped together. Abbreviations: LDH, lactate dehydrogenase; CRP, C-reactive protein; SD, standard deviation.

**Table 3:** Parameters predictive for sphincter-preserving surgery in multivariable analysis.

Parameter	OR	95% CI	*p*-Value
Sex			
Female	1		
Male	0.958	0.482–1.905	0.904
Age			
≤60	1		
>60	2.475	1.249–4.903	0.009
Karnofsky performance status			
≤80%	1		
>80%	0.556	0.1175–1.770	0.321
Clinical T-size			
cT 1–3	1		
cT 4	3.759	1.214–11.641	0.022
Histopathological subtype			
Adenocarcinoma	1		
Adenocarcinoma (mucinous)	2.198	0.624–7.741	0.220
Tumor grade			
G1	1		
G2	1.899	0.623–5.785	0.259
G3	14.067	1.896–104.376	0.010
Hemoglobin	0.992	0.819–1.202	0.936
Absolute neutrophil value	0.875	0.707–1.082	0.218
CRP value (mg/L)			
≤5	1		
>5	2.544	1.314–4.926	0.006
Neutrophil-to-lymphocyte ratio			
≤3	1		
>3	1.392	0.647–2.994	0.397
Platelet-to-lymphocyte ratio			
<150	1		
150–300	1.084	0.533–2.204	0.823
>300	0.464	0.106–2.037	0.309

Abbreviations: cT-size, clinical tumor size; CRP, C-reactive protein; OR, odds ratio; CI, confidence interval.

## Data Availability

The data presented in this study are available on request from the corresponding author. The data are not publicly available due to ethical restrictions.
